# Seasonal forecasting of green water components and crop yields of winter wheat in Serbia and Austria

**DOI:** 10.1017/S0021859617000788

**Published:** 2017-12-11

**Authors:** B. Lalić, A. Firanj Sremac, L. Dekić, J. Eitzinger, D. Perišić

**Affiliations:** 1 Faculty of Agriculture, University of Novi Sad, Dositej Obradovic Sq. 8, 21000 Novi Sad, Serbia; 2 Republic Hydrometeorological Service of Serbia, Kneza Višeslava 66, 11000 Belgrade, Serbia; 3 Institute of Meteorology, University of Natural Resources and Life Sciences, Gregor Mendel Str. 33, A-1180 Vienna, Austria; 4 Faculty of Sciences, University of Novi Sad, Dositej Obradovic Sq. 4, 21000 Novi Sad, Serbia

## Abstract

A probabilistic crop forecast based on ensembles of crop model output (CMO) estimates offers a myriad of possible realizations and probabilistic forecasts of green water components (precipitation and evapotranspiration), crop yields and green water footprints (GWFs) on monthly or seasonal scales. The present paper presents part of the results of an ongoing study related to the application of ensemble forecasting concepts for agricultural production. The methodology used to produce the ensemble CMO using the ensemble seasonal weather forecasts as the crop model input meteorological data without the perturbation of initial soil or crop conditions is presented and tested for accuracy, as are its results. The selected case study is for winter wheat growth in Austria and Serbia during the 2006–2014 period modelled with the SIRIUS crop model. The historical seasonal forecasts for a 6-month period (1 March-31 August) were collected for the period 2006–2014 and were assimilated from the European Centre for Medium-range Weather Forecast and the Meteorological Archival and Retrieval System. The seasonal ensemble forecasting results obtained for winter wheat phenology dynamics, yield and GWF showed a narrow range of estimates. These results indicate that the use of seasonal weather forecasting in agriculture and its applications for probabilistic crop forecasting can optimize field operations (e.g., soil cultivation, plant protection, fertilizing, irrigation) and takes advantage of the predictions of crop development and yield a few weeks or months in advance.

## Introduction

Both plants and the atmosphere are non-linear dynamic systems. An important feature of such systems is that even small perturbations of the initial conditions can cause the system to evolve along significantly different paths (Lorenz 1963). In the case of plants and the atmosphere, the exact values of the initial conditions are unknown.

Even Charney's first numerical weather prediction (NWP) (Charney *et al.* 1950) was a deterministic one, and the impact of uncertainties in the initial conditions on the NWP outputs soon became an important topic of short-range and, particularly, long-range (monthly and seasonal) weather forecasting. A comprehensive overview of the building of the framework for the present ensemble weather forecast systems can be found in Lewis (2005). Currently, an ensemble seasonal weather forecast (SWF) is assimilated either from an ensemble of atmospheric models run with the same initial and boundary conditions or from an ensemble of multiple runs of one atmospheric model with perturbed initial conditions.

Following the ensemble forecast concept in meteorology, modelling of plant development can achieve the same non-deterministic dimension through the use of an ensemble of models with the same initial conditions (related to weather, soil and plants) (Higgins 2015; Higgins *et al.* 2016), multiple runs of the same model with perturbed initial conditions (a strategy that is often applied for model parameterization or sensitivity analyses of input parameters (Kersebaum 2011; Eitzinger *et al.* 2013*a*), or a combination of these strategies. The different initial conditions strategy can be performed using an ensemble weather forecast as the input weather data for a crop model with or without perturbed soil and crop characteristics. Considering that the energy and water balance models are modules of dynamic crop models, the described procedure results in an ensemble of estimates of the energy and water balance components, as well as the green water (GW) components (precipitation and evapotranspiration) (Hoekstra *et al.* 2011).

The ensemble of estimates of the weather conditions and crop model outputs (CMO) can be transformed into probabilistic forecasts in order to measure the uncertainties in weather (Tennekes 1988) and crop forecasting (Higgins 2015). The distribution functions arising from an ensemble (Wilks 2002; Jewson *et al.* 2004) convey additional information about the forecast variable, which is often used for scientific studies and practical applications (Bröcker & Smith 2008). Different approaches for supplying SWF information to crop simulation models can be found in Hansen & Indeje (2004) and Baigorria *et al.* (2008).

Agricultural production, as a weather-dependent human activity, can benefit most from the application of SWF (Meinke & Stone 2005; Sivakumar 2006), especially in combination with agrometeorological models, which provide better tailored information for the specific applications of SWF, such as for yield forecasting or pest warnings for farmers (Hansen 2005). Weather-sensitive events (the start of specific crop growth stages, the presence and intensities of frosts and droughts, etc.) and farming decisions rely heavily on the accuracy of short- and long-range weather forecasts. However, monthly and SWFs in Europe are far from achieving systematic use for farms and, by extension, practical services (Calanca *et al.* 2011). A review of SWF application-related papers and services suggests that the end-user community is not well informed about the features, uncertainties, applicability and means of using SWF products. This may have resulted from: (a) the complex procedures for obtaining forecast products in numerical form and (b) the current state of the methodology for the application and validation of long-range weather forecasting for agrometeorological purposes. Some significant steps forward were achieved by the DEMETER project (Development of a European Multimodel Ensemble System for Seasonal to Interannual Climate Prediction; Palmer *et al.* 2004) and the more recent ENSEMBLES project, which intended to develop an ensemble prediction system (EPS) for climate change based on the predominant state-of-the-art high-resolution global and regional Earth System models (Doblas-Reyes *et al.* 2009; Weisheimer *et al.* 2009).

At the regional level, SWF has received particular attention in those regions affected by the El Niño-Southern Oscillation (ENSO) phenomenon (Subbiah & Kishore 2001; Meinke & Stone 2005) and those especially vulnerable to extreme weather events and climate change impacts (Huda *et al.* 2004; Marletto *et al.* 2005; Detlefsen 2006; Harrison *et al.* 2007; Semenov & Doblas-Reyes 2007; IPCC 2012). Advance warnings of droughts, flooding, heat waves, early and late frost events, etc., based on long-range weather forecasts, especially within the timescales of 1–6 months, can make agricultural production more sustainable (Ogallo *et al.* 2000; Fraisse *et al.* 2004; Hansen 2005; Hansen *et al.* 2006; Andre *et al.* 2010; Das *et al.* 2010; Ferrari 2010).

In Central Europe, significant increases in drought and heat events are expected under climate change scenarios (Trnka *et al.* 2011, 2014; Thaler *et al.* 2012; Eitzinger *et al.* 2013*b*), supporting the increasing potential of applications of SWF in the next decades. The operational use of SWF can significantly affect: (a) farm operations (planting, harvesting and soil cultivation timing; fertilizer/pesticide application; crop selection; and seed purchases); (b) irrigation water demand and crop water productivity; and (c) improved knowledge related to crop growth rate, storage needs, transport requirements, insurance, marketing and consumer demand (Davey & Brookshaw 2011).

The present study investigates the capabilities of SWF to provide possible ranges of values of meteorological elements (temperature and precipitation), CMOs and green water footprint (GWF). Particular attention is devoted to evapotranspiration as a GW component and yield as a GW-related CMO. The hypothesis is that seasonal crop model simulations, particularly ensemble ones, should benefit from SWF since, for many physiological (growing) processes, the sum of temperatures above a certain threshold and accumulated precipitation during a season can be good performance predictors. For agricultural purposes, the appearance and duration of extreme temperatures or temperatures above/below thresholds during the period of interest (i.e., the phenological phase) are important, regardless of the exact date. Additionally, analysis of an ensemble probability distribution offers a measure of the uncertainty of the obtained results as well as the possibility of testing the role of crop models as probability function filters. The current study tests this hypothesis and assumption and provides a methodology for the further use of SWF in agricultural production.

The objectives of the present study are as follows: (1) to perform seasonal crop forecasting by using deterministic and ensemble weather forecast as the input weather data for a crop model without perturbing soil or crop characteristics (as described above); (2) to assess the ensemble forecast's ability to provide a narrow range of feasible CMOs and the associated GWF of the crops (Mekonnen & Hoekstra 2010; Gobin *et al.* 2017) based on the ensemble spread (Toth *et al.* 2003); (3) to test seasonal CMO and GWF forecasting by comparing the deterministic and ensemble estimates with the results obtained using observed weather data; and (4) to analyse the CMO and GWF ensemble estimates distributions and evaluate them using ignorance scores (Good 1952; Roulston & Smith 2002).

The intention of the present paper is to present a methodology for (a) the implementation of one scenario for obtaining ensemble estimates of CMO and GWF and (b) the analysis of the obtained results. As a case study, the SIRIUS crop model (Semenov & Porter 1995; Jamieson *et al.* 1998) and SWF (deterministic and ensemble) provided by the European Centre for Medium-range Weather Forecast (ECMWF) are used to provide a seasonal forecast of the CMO and GWF for winter wheat in Austria and Serbia.

## Materials and Methods

### Study area

**Fig. 1.**
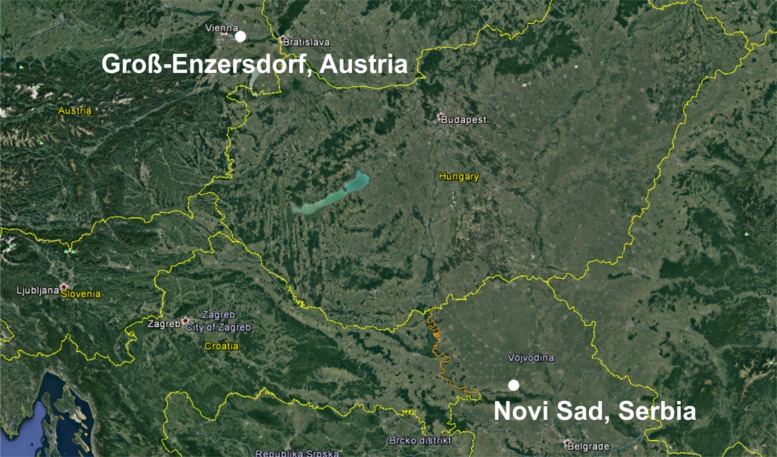
Map of selected locations in Serbia (Novi Sad) and Austria (Groß-Enzersdorf).

Two locations were selected (Fig. 1) from the most important agricultural production areas in Austria (Groß-Enzersdorf (GE) – 48°12′N, 16°33′E, 148 m asl) and Serbia (Novi Sad (NS) – 45°15′N, 19°50′E, 84 m asl); both locations have grown permanent winter wheat crops for many decades. In addition, both locations are situated on the flat terrain of the southern and south-western parts of the Pannonian lowland, although Groß-Enzersdorf's weather is strongly influenced by the presence of the Alps mountain range to the west and southwest. The typical climate of the study areas is continental or moderate continental, with mean annual temperatures of 11.5 °C in NS and 10.8 °C in GE and mean annual precipitation of 647 mm in NS and 550 mm in GE for the reference climatological period 1981–2010. The mean annual temperature during the winter wheat-growing period (October–July) was 10 °C in NS and 9.1 °C in GE, while the mean annual precipitation was 534 mm in NS and 426 mm in GE. An important feature of the continental-type climate in these areas is the high variability in temperature and precipitation, especially during the spring, which is often expressed by extreme weather events and hot and dry conditions during summer (Müller 1993; Lalic *et al.* 2013).

### Data

For the purposes of the present study, the following daily meteorological data were used: daily maximum (*T*_max_) and minimum (*T*_min_) air temperatures, daily average relative humidity (RH), daily sum of global solar radiation (Rg), wind speed (*v*) and 24-h accumulated precipitation (*P*). The selected variables represent the full set of meteorological input data commonly used in crop modelling. Since the start of active winter wheat growth in spring at both locations is, on average, during March, 1 March is set as the start date in the seasonal time series. To avoid any lack of data, the dataset was extended 1 month after the usual end of the winter wheat-growing period and the selected time series (2006–2014) include data from 1 March to 31 August.

#### Observed data

Historical records of the selected meteorological data for the NS and GE weather stations were obtained from the national weather service (Hydrometeorological Service of the Republic of Serbia and Central Institute for Meteorology and Geodynamics of Austria, or ZAMG). Due to a lack of global solar radiation measurements for both locations, this variable was calculated using Prescot's empirical formula (Trnka *et al.* 2005).

#### Seasonal weather forecast data

The long-range or seasonal forecasts follow the same approach as the NWPs in an attempt to provide information about climate conditions over the next few months or seasons. While weather forecasting is the prediction of continually changing conditions in the atmosphere, a seasonal forecast is a summary of statistically estimated weather events during that season. Numerical weather prediction is extremely sensitive to slight differences in the initial conditions, which can lead to the development of different processes in the atmosphere and can subsequently reduce the accuracy of the forecast after a 10-day period.

Over the past decade, the European Centre for Medium-range Weather Forecast has developed a system for ensemble seasonal forecasting based on the same system of hydrodynamic equations used in medium-range forecasting. In the developed system, perturbations were used to create the initial conditions for the ensemble run. It should be emphasized that a seasonal forecast cannot predict daily variations in meteorological elements at specific locations months in advance because of the chaotic nature of the atmosphere but can provide a possible range of these elements. The seasonal forecast system of ECMWF begins with 10 ensemble members (EMs) in 2006 for 6 months and progresses to 50 EMs in 2014 for a 7-month forecast.

The present study used ECMWF seasonal forecast products, starting on 1 March for all available years, and the EMs in the MARS (Meteorological Archival and Retrieval System). The 24-h average values for several parameters from the start to the end of the forecast period were used. Two separate locations were considered: NS (45°15′N, 19°50′E) and GE (48°12′N, 16°33′E). The resolution of the seasonal ensemble forecast data was 0·5° × 0·5°. From those fields, the geographically averaged values were extracted from the four nearest numerical points. Because of the specific terrain and steep hills near Groß-Enzersdorf, the selection did not match the observational data well. A comparison of the real topography with the static field of model orography for a given horizontal resolution helped to select the best option, which was one of the nearest numerical points to the southeast.

Importantly, the deterministic element of the EPS is the deterministic – control forecast. A control forecast, or a control run (CR) in terms of EPS, is a forecast model run without any perturbations of the initial conditions to analysis. Providing the initial conditions for the control analysis consists of collecting observations and interpolating data from irregularly spaced locations to the model grid and its objective analysis.

To test the efficacy of SWF in crop and GWF modelling, two datasets based on the CR and EMs were designed for the entire period of interest (1 March to 31 August) during the selected 9-year period. The results of the crop model simulations and GWF calculations using EMs were averaged in order to obtain ensemble averages (EA) (Anderson *et al.* 2007). The results obtained using the observed and control run datasets are denoted as OB and CR, respectively.

### Crop model simulations and green water footprint calculations

The dynamic crop growth simulation model SIRIUS was run using the SWF and observed weather data for NS (Serbia) and GE (Austria). SIRIUS has previously been calibrated and validated for the agroecological conditions of the Vojvodina (Serbia) region (Lalic *et al.* 2013) and was applied in the present study to produce ensemble CMOs using SWF. Accumulated evapotranspiration (AccET) during the growing season, maximum water deficit (MaxD), anthesis day (AnthD), maturity day (MatD) and grain yield (Yield) were the simulated outputs for the selected locations in Serbia and Austria given a chernozem soil and using the OB, CR and EA datasets.

The selected winter wheat cultivar for the simulation study was ‘Balkan’, for which a crop model was successfully validated in a previous study (Lalic *et al.* 2013). In addition to the soil characteristics of the chernozem soil, data related to the timing and number of management operations, variable characteristics and phenological dates were recorded for the selected season. Because the middle of October is the typical time for sowing winter wheat in both the Serbian and Austrian test regions, 15 October was set as the time of sowing for all runs. A typical management scenario was used, with no irrigation and only three fertilizer applications: (i) before sowing (10 October) – 50 kg N/ha, (ii) at the end of the winter (2 February) – 55 kg/ha and (iii) during spring (4 April) – 40 kg/ha.

The GWF of a crop is calculated using the following method established by Mekonnen & Hoekstra (2010):
1
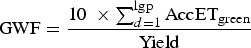

where the subscript ‘green’ indicates rainfed conditions and *l*gp is the length of the growing period. In Eqn (1), AccET is in mm/day, and yield is in t/ha.

### Verification statistics of the ensemble forecast

#### Ensemble forecast and control run *v.* observations

The verification methodology, based on the calculation of the root mean square error (RMSE) and ensemble spread (SPRD) (Toth *et al.* 2003), was used to evaluate the ensemble-based temperature (*T*_max_ and *T*_min_) and precipitation (P) forecasts during the period of interest (1 March–31 August) for the selected locations. The RMSE of the ensemble average (RMSE^EA^), which is a measure of the difference between the forecast and the observation, was calculated as follows:
2
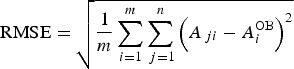

where *A*_*ji*_ is the value of variable *A* for the *i*th element of the sample (day in this case) and for *j*th ensemble member, 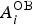 is the observed value of *A* on the *i*th day, *m* is the sample size (182 days) and *n* is the ensemble size. Commonly, the ensemble average 
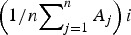
 of variable A for the *i*th element of the sample is denoted with 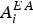. The SPRD, which represents the uncertainty of the ensemble, is defined as follows:
3
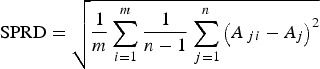

It is important to note that a small SPRD does not necessarily imply a high skill of a forecast but can be a good indicator of high predictability.

This methodology was partially adapted for the correlation of CMO and GWF EMs for each year, and the corresponding yearly realizations were calculated using the observed weather data (OB). Since each EM was equally probable, the RMSE, as a measure of CMO and GWF forecast accuracy, was calculated for each year, comparing the values of CMOs and GWFs calculated using the EMs, *Y*_j_, and observed data, *Y*^OB^, as follows:
4
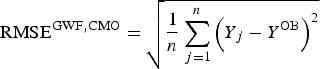

The deviation of the ensemble forecast from the mean is an important attribute of the ensemble-based CMO and GWF calculations. Therefore, the SPRD for each year was calculated using the following formula:
5
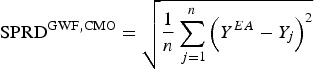

Comparisons of Eqns (2) and (3) and Eqns (4) and (5) show that an ideal SWF will have RMSEs and SPRDs with the same magnitude since, in that case, the forecast value is equal to the observed value for each EM. Accordingly, the simulation obtained using the ensemble forecast is more realistic when the RMSE and SPRD values are similar.

To assess the deviation of the CMOs and GWFs obtained using SWF from the observation-based results, the RMSE and standard deviation, *σ*, were calculated using the average values of CMOs and GWFs across all EMs as well as the values obtained using the CR dataset for each year over the period of 2006–2014.

From the procedures described by Pielke (1984), the simulation can be considered more realistic if (a) the RMSEs obtained using the simulated data (RMSE^CR^ and RMSE^EA^) are less than the standard deviation of the observed values (σ_OB_) and (b) the standard deviation, σ values of the CMOs and GWFs calculated using the forecasted data (σ_CR_ and σ_EA_) are similar to that obtained when using the observed weather data, σ_OB_. The RMSE was calculated for both the EM and CR datasets for the chosen decade since it provides a good overview of the datasets, with large errors weighted more than many smaller errors (Mahfouf 1990).

To test the interannual variability of the SWF-based products and their ability to match the counterpart values in the observations, the coefficient of variability, *c*_v_, of the selected CMO and GWF was calculated using the EM and CR datasets over the 2006–2014 period and was compared with the results obtained using the OB data.

#### From ensemble forecast to probability distribution

A comprehensive overview of the theory behind the transformation of an ensemble of estimates into a distribution function, e.g. a probabilistic forecast, can be found in Bröcker & Smith (2008) and Siegert *et al.* (2016). An important feature of a probabilistic forecast is its performance measure or scoring rule (Gneiting & Raferty 2007). The ignorance score is a commonly used method that is defined by the following scoring rule (Roulston & Smith 2002):
6
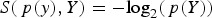

where *p*(*Y*) is a unitless probability density function of the verification value of variable *Y*.

The ignorance score quantifies the performance of an ensemble forecast, by measuring the logarithm of probability density (which, in the present paper, is the Gaussian kernel because only normally distributed variables are considered) of the normalized value (*Z*-value) of the outcome. Lower ignorance scores indicate more skillful forecasts. The Normal distribution obeys the 68-95-99·7 rule; thus, the ignorance score can be expected to be <2·04 with probability 0·68, <4·21 with a probability of 0·95, and >7·81 with a probability of 0·3. Therefore, if the ignorance is <2·04, the model's skill can be considered as very good, and if it is >7·81, the model is not adequate.

In the present study, a score was applied where *Y* is a value calculated using the OB dataset. From Eqn (6), a decrease in the ignorance score corresponds to a better simulation. In the present paper, as a first step in the analysis of the results obtained, only an ensemble of estimates that have Gaussian (Normal) distributions is considered (the Shapiro–Wilk test and *Q*–*Q* plot were used to determine whether the distribution is normal). To compare the scores obtained for the different variables and those coming from different normal distributions, the variables were normalized and a *Z* score used. The *Z* score is introduced by replacing the variable of interest with *Z* = (*Y−μ*)/*σ*, where *μ* and *σ* are the mean and the standard deviation of the ensemble. Consequently, the probability density function becomes a standard Gaussian density ensemble as follows:
7
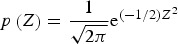


## Results

### Seasonal forecast of air temperature and precipitation

**Fig. 2.**
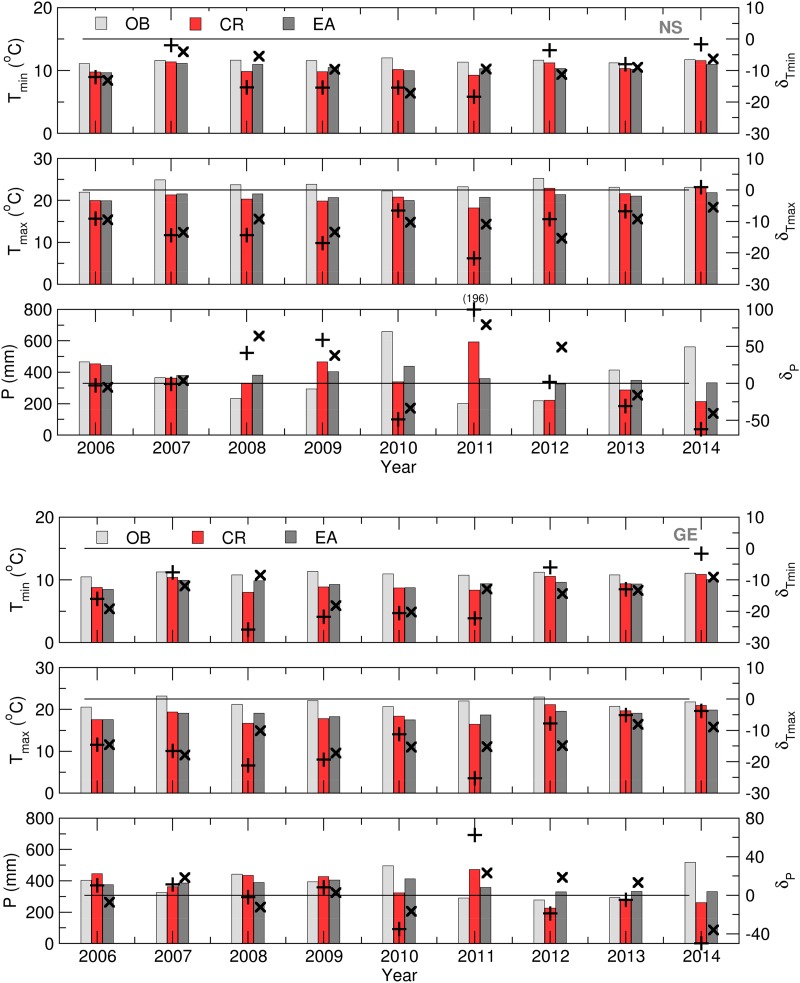
*T*_min_, *T*_max_ and P for 1 March–31 August: the average values (bars) and relative deviations (‘+’, CR; ‘×’, EA) obtained using the OB, CR and EA datasets for NS (up) and GE (down) for 2006–2014.

The results obtained by comparing the average maximum and minimum temperatures, accumulated precipitation and the relative deviations (Fig. 2) obtained using OB, CR and EA can be summarized as follows: (a) The *T*_min_ forecast based on EA slightly differs from that of the CR in Groß-Enzersdorf. The deviation from the observations was more pronounced in GE than in NS for both the CR and EA datasets. (b) The *T*_max_ forecast based on EA in both locations is underestimated every year. (c) The accuracy of the P forecast significantly varied between seasons at both locations, while the results obtained using EA were closer to the observed results. The accuracy of the *P* forecast significantly varied between seasons at both locations, while the results obtained using EA were closer to the observed results. In NS, in 2006 and 2007, the difference between CR and EA were negligible (2006: *δ*_CR_ = −2·75%; *δ*_EA_ = −5·18%; 2007: *δ*_CR_ = −0·68%; *δ*_EA_ = 3·95%), while during the rest of the analysed period (5 of the 7 years), the relative deviation of EA was less than that of CR. In GE, in 2013, the difference between CR and EA was just 8·77% (in 2009 5·45%), while in 5 of the 8 years, the EA forecast produced a lower relative deviation with respect to observations. The differences between the observations and the forecasts were particularly pronounced in 2010 and 2014 at NS, mainly because precipitation was greatly underestimated in 2010 and 2014 (Fig. 2). During the period of 1 March to 31 August in 2010, the recorded precipitation at NS was 655 mm and the average annual precipitation was 647 mm. Most of this precipitation (553 mm) occurred from May to August, with monthly precipitation values that exceeded the average monthly values by >50% (in the August, precipitation was 3·6 times the long-term average). In the spring and summer of 2014, precipitation well above the climatological mean was observed, and excessive flooding occurred in Serbia. Even the ECMWF-issued accurate medium-range forecasts for that particular event were not reflected in the long-range forecast, primarily because of the long time series of the climatological values.

**Fig. 3.**
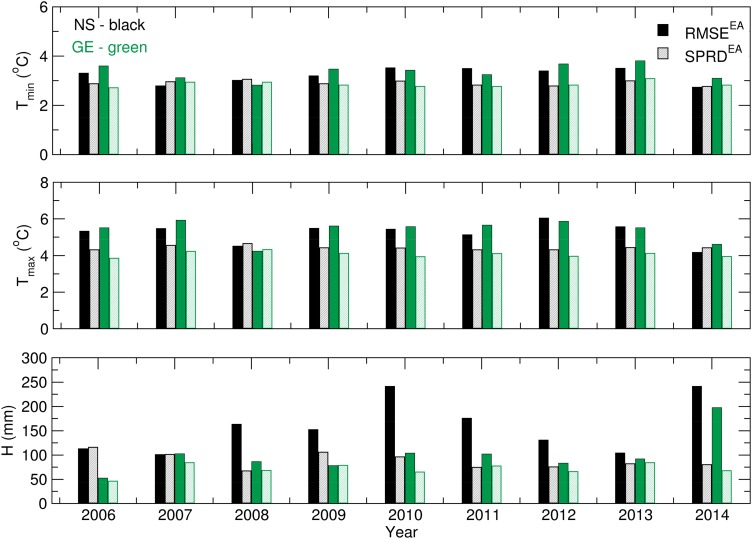
*T*_min_, *T*_max_ and *P* for 1 March–31 August: RMSE^EA^ and SPRD^EA^ values for 2006–2014.

Figure 3 presents the ensemble verification statistics for the temperature and precipitation of the NS and GE locations from 1 March to 31 August. RMSE^EA^ and SPRD were calculated using Eqns (2) and (3) for each season between 2006 and 2014 and were compared to assess the accuracy of the ensemble forecast. The presented results are summarized as follows: (a) the *T*_min_ forecast achieved nearly the same accuracy for both locations, with small variations during the period of interest; (b) the *T*_max_ forecast was slightly less realistic, and the differences between the RMSE^EA^ and SPRD values were more pronounced for Groß-Enzersdorf; (c) the ensemble skill for modelling *T*_min_ was greater than that for *T*_max_ for both locations; and (d) the P and *T*_max_ ensemble forecasts were better in GE than in NS. The significant differences between the precipitation RMSE^EA^ and SPRD for NS in 2010 and 2014 were related to the excessive rain and flooding during the vegetation period and the ensemble forecast's ability to predict extreme weather events. Additionally, the ensemble forecast had trouble predicting the precipitation in GE in 2008, when two rainy episodes with excessive amounts of precipitation were recorded: 18–21 May with 86·5 mm and 1–4 June with 65 mm. It is important to note that the rain event forecasting was accurate, but the amount of precipitation was remarkably underestimated.

### Crop model outputs and green water footprint

#### Ensemble forecast and control run *v.* observations

**Fig. 4.**
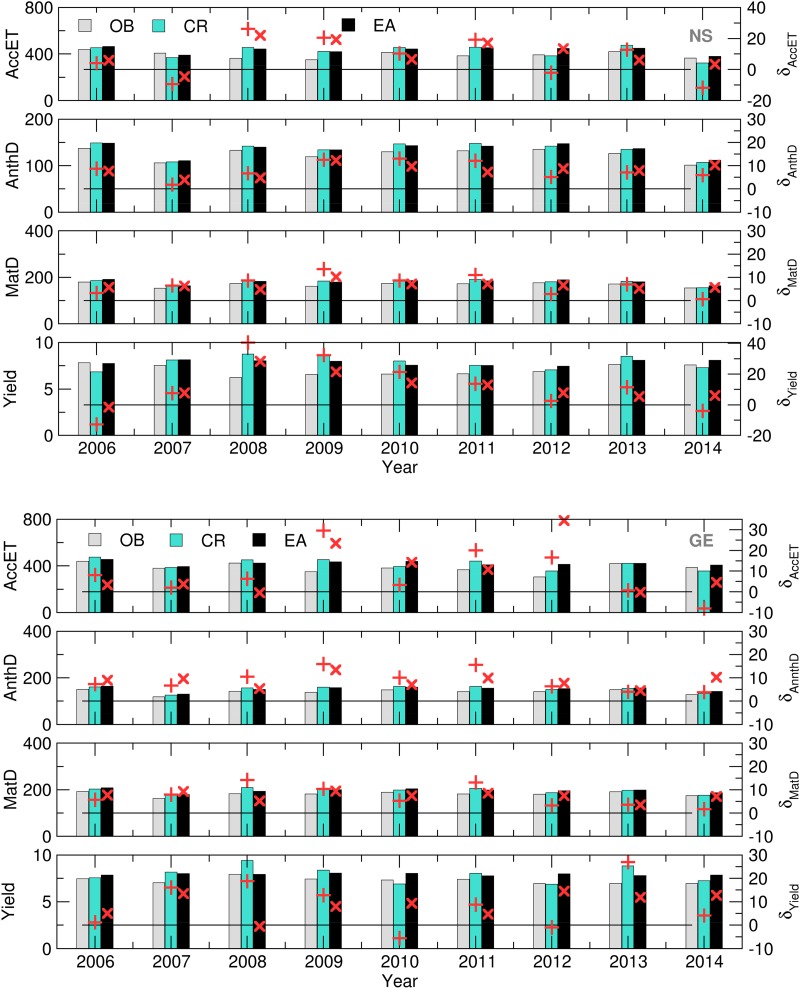
Yield (t), MatD (DOY), AnthD (DOY) and AccET (mm) (bars) and its relative deviations (‘+’, CR, ‘×’, – EA) calculated using the OB, CR and EA datasets for NS (up) and GE (down) for 2006–2014.

**Fig. 5.**
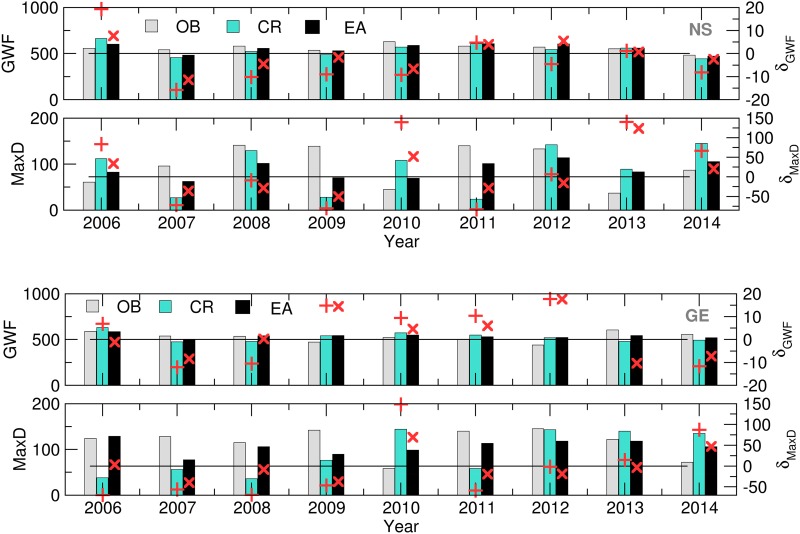
MaxD (mm) and GWF (m^3^/t) (bars) and its relative deviations (‘+’, CR, ‘×’, EA) calculated using the OB, CR and EA datasets for NS (up) and GE (down) for 2006–2014.

SIRIUS was run using the OB, CR and EM datasets as the crop model weather input data in order to obtain ensemble estimates of the selected variables. Afterwards, the GWF was calculated using Eqn (1). The CMOs and GWF obtained for each EM were averaged to obtain the CMO ensemble averages denoted with EA. The quality of the CMOs and GWFs obtained using SWF data were tested via a comparison with the OB-based CMOs (Figs 4 and 5).

The differences in simulated AnthD and MatD using EA and CR for both locations are negligible, most probably because the summation of air temperatures diminishes small underestimates and overestimates of forecast values. However, slightly better results are observed in case of AccET (7/9 in NS and 5/9 in GE), Yield (5/9 on both locations), GWF (6/9 in NS and 7/9 in GE) and MaxD (7/9 in NS and 8/9 in GE).

During the entire period of interest, it can be noted that certain overestimations of the forecasted accumulated evapotranspiration (up to 30%) and crop yields (up to 20%) with respect to OB-based simulations occurred at both locations. However, the same effect did not occur with the calculated GWF (up to 10 and 20% in NS and GE, respectively) NS. In the case of a MaxD, significant differences between the SWF (EA and CR) and OB-based results were observed. The mostly underestimated values during 2007–2009 and 2011–2012 at both locations were the result of the overestimation of precipitation and underestimation of T_max_ in the forecast *v.* the observed data. The excessive rains during the 2010 and 2014 (and part of the 2013) growing seasons were underestimated in the SWF, leading to an overestimated water deficit. However, this result proves that crop models can reproduce the input weather patterns in the MaxD calculations.

**Fig. 6.**
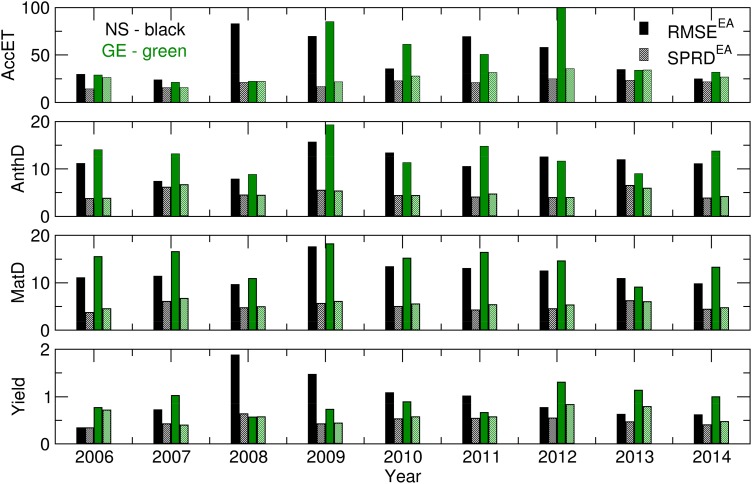
RMSE and SPRD for Yield (t), MatD (JDAY), AnthD (JDAY) and AccET (mm) calculated for 2006–2014.

**Fig. 7.**
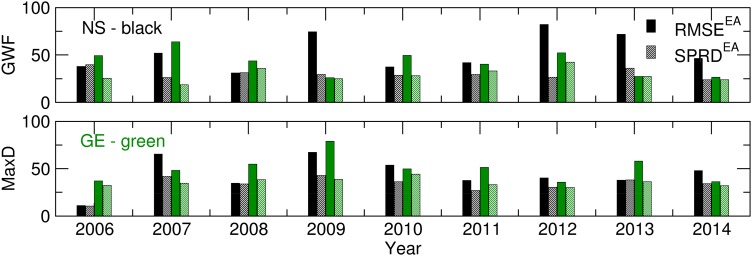
RMSE and SPRD for the MaxD (JDAY) and GWF (m^3^/t) calculated for 2006–2014.

Promising results from the ensemble crop modelling can be found in the low spread of all of the calculated Yield, AccET, AnthD, MatD and GWF values (Figs 6 and 7). The difference between the RMSE and SPRD was, to a considerable extent, in accordance with the RMSE and SPRD magnitudes and differences obtained for temperature and precipitation (Fig. 3). For example, at both locations in the 2006–2007 and 2009–2012 crop-growing periods, the high RMSEs and large deviations from the SPRD for the phenology dynamics (AnthD and MatD), particularly in Groß-Enzersdorf, corresponded to high deviations of the *T*_max_ forecast from those observed. The high RMSE values for Yield and ET in 2008 in NS are the result of high RMSE values for the precipitation of that year. However, this problem in the precipitation forecast for 2008 did not affect MaxD or GWF significantly, except in that the differences between the RMSE and SPRD for GE were more pronounced. The high RMSE and SPRD values for precipitation in 2010 and 2014, particularly at NS, and for 2008 at GE were not visible in the RMSE and SPRD values obtained for the evapotranspiration and yield because most of the precipitation producing this deviation occurred at the end of the winter wheat growing period. However, the high RMSE for evapotranspiration in 2012 at GE and for the yield in 2008 at NS cannot be explained by previously noted deviations in meteorological elements. The probable cause can be found in the seasonal forecast of the solar radiation intensity, wind speed and air humidity, i.e. the input meteorological data, for the calculation of the CMOs and GWF, which were not the subject of the current study.

#### From ensemble forecast to a probability distribution

**Fig. 8.**
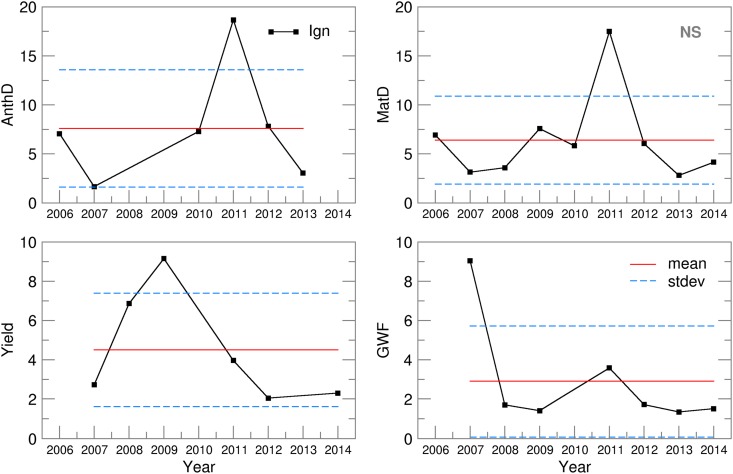
Ignorance score, standard deviation of the ignorance score and the mean ignorance score for Novi Sad.

An analysis of the probability distribution was made for all CMOs and GWF ensemble estimates. At both locations, for the study period, the following variables had normal distributions: MatD, AnthD, Yield and GWF. At both locations, in 8 of the 9 years, the OB-based values for MatD and AnthD were between the 95 and 99·7% confidence intervals (between ±2*σ* and ±3*σ* from the ensemble mean). For Yield and GWF, the results were much better, bringing the OB-based values to a much narrower interval between the 68 and 95% confidence intervals (between ±1*σ* and ±2*σ* from the ensemble mean). The ignorance score, *S*(*p*(*Y*)), was calculated for each year. Figure 8 presents the skill of the SIRIUS-based ensemble of crop model estimates, in the form of the ignorance scores for the selected CMOs and GWF for both locations.

**Fig. 9.**
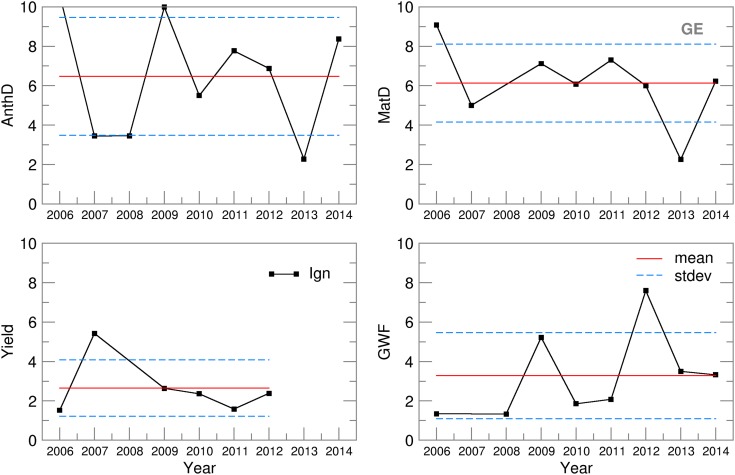
Ignorance score, standard deviation of the ignorance score and the mean ignorance score for Groß-Enzersdorf.

For NS, the ignorance scores for maturity (*S* = 6·4) and anthesis day (*S* = 6·8) forecasting lay within the mean plus/minus one standard deviation (*σ*^MatD^ = 4·5 and *σ*^AnthD^ = 5), except for 2011, which undermined the otherwise much better scores (*S*^MatD^ = 5·9, *S*^AnthD^ = 6·4, *σ*^MatD^ = 1·8, *σ*^AnthD^ = 2·5). The considerable differences between the RMSE and SPRD for MatD and AnthD in 2009–2011 (Fig. 6) could be traced only to the 2011 score. For GE, a better ensemble skill was obtained for phenology dynamics (*S*^MatD^ = 5·3, *S*^AnthD^ = 5), with much lower standard deviations (*σ*^MatD^ = 2, *σ*^AnthD^ = 3) but with higher variations over the period of interest (Fig. 9). The yield ignorance score, *S*^Yield^, at NS (3·8) was slightly greater than the value seen at GE (2·6) but also had twice the standard deviation (2·6). The low probability skills in 2008–2009 for NS and in 2007 for GE were the result of significant deviations between the ensemble estimates and yields obtained using the observed weather data, which could also be seen in the high RMSE values (Fig. 6). The GWF ignorance score, *S*^GWF^, is of the same magnitude at both locations, with similar standard deviations. The extremely low skill in 2007 at NS and in 2012 in GE could not be traced in the ensemble RMSE and SPRD results.

## Discussion

Nine years (2006–2014) of ECMWF ensemble SWF data were used as the input meteorological data for the SIRIUS to produce an ensemble of estimates of CMOs and the GWF for winter wheat. A bias correction procedure was not applied to the SWF data in this phase of the study. There are two suggested post-processing procedures that can be applied to correct model bias based on the assumption of the quasi-linear behaviour of the atmosphere, and in both cases, there will be some inaccuracy in the estimate of bias and the definition of climate (Anderson *et al.* 2007). Research on more fully calibrated products is ongoing in ECMWF and experimental calibrated products may become available for certain fields.

SIRIUS is already calibrated and validated for select locations; therefore, the model outputs obtained using the observed meteorological data were considered as being closest to the observed values.

Reviewing both the ensemble estimates and the following RMSE and SPRD values of the input weather data *v.* the output crop models and GWF data show that a straightforward signal, which can be seen in the input temperature/precipitation ensemble forecast data, is present in the crop model estimates and GWF but is less clear in years with extreme weather events, such as 2010 and 2014 at NS. For example, particularly high SPRD values and large differences between the RMSE and SPRD values for the maximum temperature in 2007 and 2009–2013 and for precipitation in the 2008–2012 period at NS can be identified in all CMOs, the GWF in 2009 and the phenology dynamics in 2009–2014. However, in the ‘extreme’ years 2010 and 2014, the SPRD for precipitation differed from the RMSE by −60 and −66·7%, respectively, while the differences for AccET (2010: −35·4%, 2014: −12·6%), Yield (2010: −50%, 2014: −34·2%), MaxD (2010: −32·9%, 2014: −28·6%) and GWF (2010: −23·5%, 2014: −48·3%) were much less pronounced. This clearly proves the first hypothesis, i.e., that ensemble crop model simulations can benefit from SWF data, even when the deviations from observations are not negligible.

A comparison between the probabilistic (ensemble) and deterministic (CR) CMOs and GWF forecasts, for both NS and GE indicates that the results obtained using the ensemble forecasts are, in general, in better agreement with the results based on observations. The advantage of ensemble prediction-based results in comparison with deterministic ones is due to the application of the ensemble forecast strategy (which copes with initial condition uncertainties by repeatedly running the NWP model using slightly perturbed initial conditions) while running a crop model and analysing an ensemble of estimates instead of one deterministic output. An important feature of the CR-based CMOs and GWF is also the high standard deviation (Table 1), which is often much higher than the standard deviation of the OB-based results. This higher standard deviation, in comparison to the OB-based results, is a result of CMOs and GWF uncertainties introduced by weather data coming from a deterministic (CR) forecast.

**Table 1.** CMO: Average values, RMSE, standard deviations, *σ* and variation coefficient, *c*_v_, for OB, CR and EA for 2006–2014

Variables	AccET (mm)	AnthD (JDAY)	MatD (JDAY)	GWF (m^3^/t)	MaxD (mm)	Yield (t/ha)
Novi Sad						
OB	392·56	124	168	558·41	97·67	7·051
CR	422·22	135	180	539·26	89·33	7·863
EA	427·46	133	178	548·93	87·72	7·825
RMSE^CR^	18·64	3·79	4·45	19·55	23·15	0·444
RMSE^EA^	16·04	3·49	3·77	10·95	10·91	0·310
*σ*^OB^	9·21	4·09	3·02	12·46	13·40	0·186
*σ*^CR^	16·37	5·12	3·89	22·50	15·80	0·225
*σ*^EA^	9·16	4·44	3·17	16·18	5·77	0·086
*c*_v_^OB^	0·26	0·37	0·20	0·25	1·52	0·29
*c*_v_^CR^	0·43	0·42	0·24	0·46	1·97	0·32
*c*_v_^EA^	0·24	0·37	0·20	0·32	0·73	0·12
Groß-Enzersdorf						
OB	384·22	139	182	528·74	116·22	7·268
CR	415·89	152	195	526·22	91·78	7·935
EA	417·34	150	194	529·60	104·07	7·899
RMSE^CR^	16·53	4·64	5·09	23·58	22·66	0·326
RMSE^EA^	16·92	4·10	4·54	15·66	10·91	0·235
*σ*^OBS^	12·95	3·30	2·85	16·60	9·71	0·104
*σ*^CR^	13·84	4·25	3·83	16·50	15·00	0·274
*σ*^EA^	5·93	3·24	2·73	7·76	5·00	0·033
*c*_v_^OB^	0·37	0·26	0·17	0·35	0·93	0·16
*c*_v_^CR^	0·37	0·31	0·22	0·35	1·82	0·38
*c*_v_^EA^	0·16	0·24	0·16	0·16	0·52	0·05

CMO, crop model outputs; AccET, accumulated evapotranspiration; AnthD, anthesis day; MatD, maturity dayGWF – green water footprint; MaxD, maximum water deficit; Yield, grain yield; OB, observed; CR, control run; EA, ensemble averages; RMSE, root mean square error; *σ*, standard deviation; *c*_v_, coefficient of variability.

The present study results clearly indicate that, during the entire period of interest, at both locations and for all CMOs and GWF, the SPRD values were low. This underlines the very good performance of the model, despite the relatively high RMSE values and large differences between RMSE and SPRD because of the systematic error in the simulations that can be assumed to have been introduced. It is well known that systematic errors are far easier to remove than random ones. The more locations in a selected region can be used to identify the possible sources of error in the local/regional impacts (parameterization of orography, surface model parameterizations, etc.) or large-scale impacts (related to the model packages for radiation, the advective scheme used, etc.). For example, the slightly higher deviations from the observations sometimes observed at GE are partly related to the position of the weather station. Although both stations are located on the margins of the Pannonian lowland, the GE location is in the vicinity of the Alps mountain range to the west and is affected by the associated climate gradients, especially with regard to precipitation and other water balance parameters (BMLFUW 2003). The influence of orography on NWP is a well-known problem. Because every model uses the observed orography interpolated to the model's horizontal resolution, some important characteristics, such as steep hills that can cause convection and heavy rain, are reduced or amplified numerically. However, it is important to stress one significant feature of the ensemble forecast with respect to the ensemble spread. Namely, if an ensemble forecast provides a narrow enough range of values for the variable of interest, one can assume that its ability to predict daily temperature variations, precipitation events or even yield, for example, is significant. However, sometimes large spreads in the ensemble forecasts leave windows of opportunity for the assessment of extreme weather events and low-probability atmospheric processes with significant impacts, particularly in the transition seasons and under weather conditions associated with, for example, high summer temperatures and convective processes, particularly in the spring and summer. While the ensemble spread compared with the RMSE of the ensemble reflects the model accuracy, the ignorance score indicates the skill of the ensemble forecast, i.e., it is a measure of the forecast ‘goodness’. The first results related to the probabilistic crop forecast were obtained using only a Gaussian distribution and ignorance score and indicate quite uniform forecasting skills (results within the interval of the mean ±*σ*) for both locations for the phenology dynamics, yield and GWF. The lowest average ignorance score, i.e., the highest forecasting skill, is obtained for GWF, and the value for yield is only slightly higher, which is a very promising result. However, the accumulated evapotranspiration and soil water deficit, in general, did not pass the normal distribution test, and the ignorance score, which uses the assumption of the normality of the probability distribution, is therefore not relevant for these variables. It is concluded that one should look for a more adequate probability distribution model for these variables.

## Conclusions

The present paper describes the implementation and output statistics analysis of ensemble crop and GW forecasting. It also reports the first implementation of this type of analysis, which studied winter wheat using SIRIUS. The presented simulation results and verification statistics, particularly those related to the GW components (precipitation and evapotranspiration), yield and GWF, allow the reader to (a) assess the capacity of the ensemble forecast to offer a sufficiently narrow range (when it is possible and favourable) of the possible realizations of the selected variables; (b) identify the differences between the ensemble and deterministic weather forecast (CR) applications; (c) assess the uncertainties in the ensemble estimates, i.e. the probabilistic forecast application for the selected CMOs and GWF; and (d) understand the ability of SWF to reproduce the real inter-annual variability in the CMOs and GWF on long-term scale.

In the case of winter wheat, the seasonal ensemble forecasting results obtained for phenology dynamics, yield and GWF offer a narrow range of estimates. The exceptions are extreme weather events (such as precipitation in 2014, which resulted in flooding), when the forecasted weather data commonly underestimate the observational data and when the crop models cannot reproduce crop responses (Lalic *et al.* 2014). For the operational stage, the results of the presented research can be used by producers and other decision-makers in planning the timing of farm operations and spraying (phenology dynamic forecasting), irrigation scheduling (MaxD, GWF and AccET, forecasting) and fertilization (phenology dynamics and yield forecasting).

Further plans related to SWF applications include the use of more fully calibrated ECMWF products and the use and testing of some post-processing and calibration methods. The next steps in crop ensemble forecasting using one deterministic crop model should entail the use of ensemble weather forecasts as the input weather data, with fixed soil and crop characteristics, for models of summer crops, water management and irrigation planning and phenology dynamics of orchards (i.e., grapes and fruits) for frost risk warning and frost protection. The next level in ensemble crop forecasting will be reached through the use of ensemble weather forecasts with perturbed soil and/or crop characteristics.
